# Measurement of Functional Brain Network Connectivity in People with Orthostatic Tremor

**DOI:** 10.3390/brainsci14030219

**Published:** 2024-02-27

**Authors:** Connor J. Phipps, David Whitney, James Shou, Diego Torres-Russotto, David E. Warren

**Affiliations:** Department of Neurological Sciences, University of Nebraska Medical Center, Omaha, NE 68198, USA; connor.phipps@unmc.edu (C.J.P.); david.whitney@inova.org (D.W.); james.shou@rochesterregional.org (J.S.); diego.torres@baptisthealth.net (D.T.-R.)

**Keywords:** orthostatic tremor, tremor, resting-state functional connectivity, transcranial magnetic stimulation

## Abstract

Orthostatic tremor is a rare movement disorder characterized by a sensation of unsteadiness and leg tremor while standing. It has been hypothesized that the disorder is attributable to dysregulation of a central oscillatory network in the brain. This putative network includes primary motor cortex, supplementary motor area, cerebellum, thalamus, and pontine tegmentum. We studied this brain network by recording resting-state functional MRI data from individuals with orthostatic tremor. For each participant, we measured resting-state functional connectivity using a seed-based approach. Regions of interest included were components of the putative central oscillatory network and a primary motor thumb region (identified via transcranial magnetic stimulation). A non-central oscillatory network region of interest—posterior cingulate cortex—was included for comparative analysis of a well-characterized intrinsic network, the default mode network. Demographic information, medical history, and tremor characteristics were collected to test associations with functional connectivity. For normative context, data from the 1000 Functional Connectomes Project were analyzed using an identical approach. We observed that tremor and demographic variables were correlated with functional connectivity of central oscillatory network components. Furthermore, relative to healthy comparison participants, patients with orthostatic tremor exhibited qualitatively different patterns of cerebellar resting state functional connectivity. Our study enhances the current understanding of brain network differences related to orthostatic tremor and is consistent with a hypothesized selective decoupling of cerebellum. Additionally, associations observed between functional connectivity and factors including medical history and tremor features may suggest targets for treatment of orthostatic tremor.

## 1. Introduction

Orthostatic tremor (OT) is a rare movement disorder characterized by sensations of imbalance while standing and a 13–18 Hz synchronous tremor [1,2]. Comorbidities include mild ataxia, anxiety-spectrum disorders, and cognitive changes [3,4,5]. Together, these changes can cause a significant negative impact on an individual’s quality of life due to fear of falling during prolonged standing and difficulty with activities of daily living. Investigators have suggested a neural generator may drive OT [6,7,8]. While this is an appealing account, substantial gaps in knowledge remain, including the locale of the suggested oscillator [9]. Studies of functional brain organization in patients with OT could address these gaps by identifying brain regions associated with OT symptoms.

**Figure 1:**
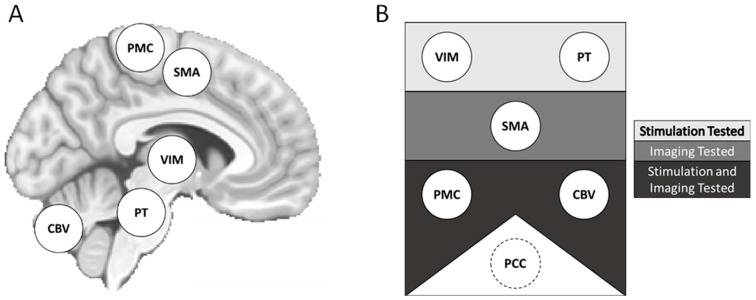
Proposed central oscillatory network (CON) components and methods of assessment. Potential elements of the proposed CON represented on a template brain (**A**). Components are also displayed by the approaches used for study (**B**). Components in the top rectangle were investigated with neurostimulatory approaches. Components in the middle rectangle, neuroimaging approaches. Components in the bottom rectangle, both neurostimulation and neuroimaging approaches. The PCC was included as a non-CON region of interest. CON = central oscillatory network. CBV = cerebellar lobule and cerebellar vermis. PMC = primary motor cortex. PCC = posterior cingulate cortex. PT = pontine tegmentum. SMA = supplementary motor area. VIM = ventralis intermedius of the thalamus.

Prior studies have described a “central oscillatory network” (CON) in the brain that may contribute to tremor in OT (Figure 1) [10]. The CON may serve to coordinate normal motor functions in healthy individuals, and dysfunction due to unidentified pathology might be associated with OT.

One approach to studying the CON involves investigation of the organization of brain networks in OT through resting-state functional connectivity (RSFC), a measure supporting characterization of intrinsic brain networks [11,12,13,14]. Measuring RSFC, particularly between putative CON elements, also allows tests of potential associations between RSFC and variables such as OT symptom severity and the OT disease process. Facilitating this network-based approach, prior reports have used multimodal techniques to identify potential CON components.

Investigators have sought to localize the CON using neuroimaging and neurostimulation. Candidate brain regions include primary motor cortex (PMC), supplementary motor area (SMA), cerebellum, thalamus, and pontine tegmentum [6,7,8,15,16,17,18,19,20]. A neuroimaging study using resting-state functional MRI (rs-fMRI) reported that individuals with OT exhibited increased connectivity between the SMA and cerebellum [6]. Meanwhile, neurostimulation of PMC, spinal cord, and thalamus have been reported to affect OT signs [7,8,15,16,20]. While prior work has identified characteristics of the CON, much remains to be determined, including the functional connectivity of its elements and potential associations with demographic and disease variables.

Here, we studied the putative CON by measuring the intrinsic functional brain connectivity of individuals with OT (*n* = 13) using non-invasive neuroimaging and neurostimulation methods. Specifically, we used rs-fMRI to measure RSFC between brain regions comprising the putative CON [7,8,15,16,20]. Regions of interest (ROIs) included primary motor cortex (PMC); supplementary motor area (SMA); cerebellum; ventralis intermedius of the thalamus (VIM); and pontine tegmentum (PT). For individuals with OT, PMC was operationalized as a left-lateralized motor thumb region localized with transcranial magnetic stimulation (TMS), a form of noninvasive brain stimulation that can assess the locale and sensitivity of the brain’s motor output regions [21]. To evaluate network integrity beyond the CON, we also measured RSFC in a well-characterized network—the default mode network (DMN)—operationalized with a seed in posterior cingulate cortex (PCC). To provide normative context, we repeated our RSFC approach with a large, publicly available neuroimaging dataset collected from healthy adults. The aim of the present study was to identify any differences of intra-CON RSFC between individuals with OT and normative expectations and find if any differences were associated with demographic or disease variables.

## 2. Materials and Methods

### 2.1. Participants

This study was approved by the University of Nebraska Medical Center (UNMC) Institutional Review Board (Protocol Code: #0389-12-EP, Date of Approval: 7 September 2012). Individuals participated after appropriate informed consent according to the Declaration of Helsinki. Informed consent was obtained from all subjects involved in the study. Participants were adults with OT aged 41–79 years (*n* = 13; 12 female, 1 male). All OT diagnoses were confirmed by a movement disorder expert (DTR) and verified with EMG. Inclusion criteria for the OT group were current clinical diagnostic criteria for primary OT [1]; no incidental findings on previous MRI; no contraindications for MRI exam; and compliance with MRI exam instructions. Exclusionary criteria were any unexplained neurological syndrome; OT Plus or Secondary OT diagnosis; and other movement disorder diagnosis. All but three participants were receiving treatment with medication for OT (gabapentin and/or clonazepam). Demographic and disease variables were collected for later analyses (Table 1).

### 2.2. MRI Data Collection

#### 2.2.1. Sites and Procedures

Data collection was carried out at UNMC. The MRI instrument was a Philips 3 Tesla Ingenia scanner with a 32-channel head coil. Excess space within the head coil was filled with foam padding to limit head motion. Furthermore, all participants were instructed to remain still and keep their eyes open during functional scans.

#### 2.2.2. Scanning Parameters

Our MRI protocol was adapted from the Adolescent Brain Cognitive Development (ABCD) study [22]. Structural T1-weighted scan sequences had the following parameters: TR = 6.31 ms; TE = 2.9 ms; flip angle = 8°; slice thickness = 1 mm; slices = 225; FOV = 256 × 240; voxel size = 1.0 × 1.0 × 1.0 mm; acquisition time = 5 min, 38 s. Functional scan parameters were TR = 800 ms; TE = 30 ms; flip angle = 52°; slice thickness = 2.4 mm; slices = 60; FOV = 216 × 216; voxel size = 2.4 × 2.4 × 2.4 mm; multiband acceleration factor = 6; Phase encoding direction = A-P; acquisition time = 5 min. Two functional scans with these parameters were collected for a total functional imaging time of 10 min.

### 2.3. MRI Data Processing

#### 2.3.1. Structural Data

T1-weighted data were converted from DICOM to NIFTI format via the dcm2niix application [23]. ANTs software version 2.1.0 was used for skull-stripping and bias correction of structural scans [24]. Bias-corrected data were submitted to the FreeSurfer processing pipeline (version 5) for segmentation and generation of white matter and cerebrospinal fluid masks for functional data analysis [25,26]. Non-linear spatial normalization of each participant’s T1 data to a common template space was carried out with ANTs software.

#### 2.3.2. Functional Data

Functional data were converted from DICOM to NIFTI format prior with the dcm2niix application version 1.0 [23]. Data processing was implemented as a workflow using AFNI software version 20.2.18 [27,28]. The workflow was as follows: (1) Despiking of echo-planar imaging (EPI) data; (2) slice-timing correction; (3) EPI coregistration; (4) EPI volume alignment to T1-weighted structural volume; (5) non-linear warp to template space; (6) masking to maintain the extent of the EPI data; (7) nuisance regression (mean white-matter signal, mean cerebrospinal fluid, global signal, realignment parameters, the first derivative of realignment parameters); and (8) spatial smoothing with a 5 mm full-width half-max Gaussian kernel (conditionally, see below). EPI data were manually reviewed by authors CP and DW for evidence of detrimental distortion, and unwarping procedures were utilized for data from four participants.

Masks for white-matter and cerebrospinal fluid were generated from the FreeSurfer processing pipeline [29,30,31]. The white matter mask was eroded twice, and the cerebrospinal fluid mask was eroded once to minimize overlap with neighboring compartments [32].

Functional runs were bandpass filtered to retain frequencies between 0.008 and 0.09 Hz. Volumes containing motion (≥0.4 mm) or large signal outliers (≥10% of voxels) were censored during regression, bandpass filtering, and later analysis. To carry out bandpass filtering without the influence of censored volumes, values were interpolated from non-censored timepoints, the bandpass filter was applied, and then the interpolated data occupying censored timepoints was re-censored [32]. Functional data processing produced data with and without spatial smoothing applied to support analysis of RSFC for ROIs of both large and small volumes. Analysis of RSFC for large volume ROIs exclusively used spatially smoothed data.

Cerebellar seeds were derived from the Wake Forest University Pick Atlas using the approach of Gallea and colleagues [6,33,34]. Anatomical masks were independent masks for each lobule and split between cerebellar hemispheres and the vermis. Cerebellar ROIs included cerebellar lobules: bilateral 4/5 (C4/5), bilateral 6 (C6), bilateral 8 (C8), bilateral 9 (C9), vermis 4/5 (V4/5), vermis 6 (V6), vermis 8 (V8), and vermis 9 (V9).

### 2.4. Transcranial Magnetic Stimulation

TMS procedures were performed at UNMC using a Nexstim 5.0 Navigated Brain Stimulation apparatus. Each participant’s T1-weighted MRI data were input into the TMS system to support stereotactic guidance. Standard motor mapping procedures were used to localize the cortical motor area corresponding to each participant’s right abductor pollicis brevis [35]. A maximum of 2 mm MRI/brain registration mismatch was allowed for motor mapping, and a successful stimulation was quantified as having the following EMG parameters: 18–26 ms latency; 100–500 μV strength. After a successful response was obtained, each participant’s individual resting motor threshold was determined. For the empirically generated TMS thumb ROI, the coordinates of each participant’s thumb stimulation were converted from the Nexstim system’s stereotactic space to template space using the non-linear ANTs warp described earlier.

### 2.5. 1000 Functional Connectomes

To provide normative context for our observations, we also analyzed data from the 1000 Functional Connectomes Project (1000FC) [36]. The 1000FC dataset, collected by Buckner and colleagues, was retrieved from the International Neuroimaging Data-Sharing Initiative [37]. The dataset was generated from participants aged 18–30 years (*n* = 198, 123 female, 75 male). Data were collected on a 3 tesla MRI instrument. The MRI protocol included T1-weighted anatomical and echo-planar imaging functional scans. Anatomical parameters were slice thickness = 1.198 mm; slices = 47; FOV = 173 × 230; base resolution = 1.2 × 1.198 × 1.198 mm. The functional parameters were TR = 3000 ms; slice thickness = 3 mm; slices = 47; FOV = 216 × 216; voxel size = 3 × 3 × 3 mm; acquisition time = 6 min.

### 2.6. Standardizing Datasets

Data from 1000FC underwent motion correction, spatial filtering with a 6-mm FWHM kernel, and rigid registration to MNI-152 template space. The dataset was submitted to the same processing described for OT data. For between-dataset comparisons of RSFC measures, OT data were downsampled from the original resolution (2 mm isotropic) to 1000FC resolution (3 mm isotropic).

Direct statistical comparison between OT and 1000FC datasets would be inappropriate due to differences between groups. Instead, the 1000FC dataset served as context for typical RSFC, and indirect/qualitative comparison to the OT group. A bootstrapping approach was implemented to support limited between-group comparisons.

### 2.7. Resting-State Functional Connectivity: ROI-ROI Analysis

Correlation between the mean timeseries of each ROI was calculated with AFNI for the OT and 1000FC datasets [27,38]. For the OT group, we determined whether significant correlations existed between ROI-ROI RSFC and the following variables: age at MRI, sex, age at OT diagnosis, age of OT onset, if medication was administered for OT, gabapentin medication for OT, clonazepam medication for OT, family history of tremor, tremor frequency (measured during EMG confirmation of OT), Unified Parkinson’s Disease Rating Scale (UPDRS), and resting motor threshold. UPDRS was applied as a measurement of tremor severity. Correlation analysis was carried out using the R software to generate Pearson correlations [39]. ROI-ROI associations were evaluated with a fixed α of 0.05 reflecting planned comparisons [6,40].

Due to the difference in size between groups, bootstrapping procedures were used to characterize the 1000FC sample. Subsets of a size equal to the OT sample were drawn from the 1000FC dataset 10,000 times. Each subset underwent analysis identical to the OT sample. The resulting statistical parametric maps (of t-values) were used to create a bootstrapped distribution. This distribution contextualized the t-values in the OT group between the ROI-ROI pairs. This produced a non-parametric rank, analogous to a *p*-value. We also applied bootstrapped observations to generate a normative connectivity matrix for the ROIs by averaging the bootstrapped samples to find parameter values between all ROI pairs for the 1000FC dataset while adjusting for the OT dataset sample size.

### 2.8. RSFC: ROI-Whole Brain Analysis

Processed rs-fMRI data were also used to measure voxelwise whole-brain RSFC associated with seed regions. A priori regions were selected based on relevance to the OT disease process [6,10,16,18,20]. Anatomically defined seed regions for the CON included cerebellar masks from the Wake Forest University Pick Atlas; left VIM; right VIM; PT; and SMA [34]. A seed in the PCC was selected to evaluate a non-CON network—the DMN. RSFC analysis was performed using AFNI in MNI-152 template space [27]. For seed information, refer to Table 2.

For OT participants, an additional ROI was empirically determined TMS to determine a PMC seed per-participant RSFC analysis. TMS data were not available for one individual in the OT group, so an ROI based on previously reported MNI coordinates for thumb region of the left motor cortex was substituted. The same MNI coordinates were used for the 1000FC dataset PMC ROI.

Statistical significance of RSFC results were evaluated via two-step procedure. First, a voxel-wise threshold was applied to identify voxels exhibiting strong responses. Second, a cluster size threshold was applied to ensure that significant voxels occurred more frequently than spatial autocorrelation would allow by chance [28,44,45,46]. Voxels surpassed a *p*-value threshold of 0.001 or were excluded from analysis. Clusterwise thresholds were set to ensure a false-discovery rate of 0.05. This threshold was empirically determined for the OT dataset using AFNI [27,44].

### 2.9. Statistics

All statistical analysis was carried out in the R statistics software (version 4.1.0) [39]. Unless otherwise stated, statistical assessments utilized a *p*-value threshold of 0.05. Statistical analysis included RSFC and derivative tests in addition to a correlation analysis of ROI-ROI RSFC and disease/demographic variables using both Pearson’s and Kendall’s correlations. This was due to the disease/demographic variables failing to pass a Kolmogrov–Smirnov test for normality. We report both correlation statistics due to the potential that tests of normality were influenced by the small sample size for our study [47,48]. An a priori power analysis for Pearson’s correlations indicated that a sample size of *n* = 13 was sufficient to detect *r* = 0.69 with 80% power (alpha = 0.05, two-tailed). We evaluated the effect size of Pearson’s correlation coefficient against the following standard cutoffs: 0.3 > *r* ≥ 0.1, small; 0.5 > *r* ≥ 0.3, moderate; *r* ≥ 0.5, large. Inspection of correlation significance post hoc revealed few cases in statistical significance of Pearson’s r and Kendall’s τ differed; thus, for rigor and concision, discussion of findings is limited to instances where both were significant.

### 2.10. Multiple Comparisons

Statistical analysis of the association between demographic and tremor variables with RSFC between CON ROIs was split into two categories: prior reported or novel associations. Existing relationships were identified from studies conducted by Gallea and colleagues or Benito-León and colleagues. Briefly, if either group reported an association between brain activity and a demographic or tremor variable, the brain region and variable were categorized as a prior identified association. A total of 15 unique prior associations were identified where the activity of a brain region was associated with a demographic or tremor variables (Table S1). For the prior reported group, each variable-ROI association was corrected for all ROI-ROI pairs (10 pairs/comparisons). For example, a significant change in brain activity in cerebellar lobule 9 was associated with disease duration so all disease duration and C9-ROI associations were corrected as a single set. All other variable and ROI-ROI RSFC associations were considered exploratory and were corrected as one group.

## 3. Results

### 3.1. Participants

The group of OT participants included significantly more females than was expected by chance (*p* < 0.05). This may have been attributable to the greater prevalence of OT in females [49,50].

### 3.2. Correlation of RSFC Patterns between Seed Regions in OT and 1000FC

#### 3.2.1. ROI-ROI Correlations

**Figure 2:**
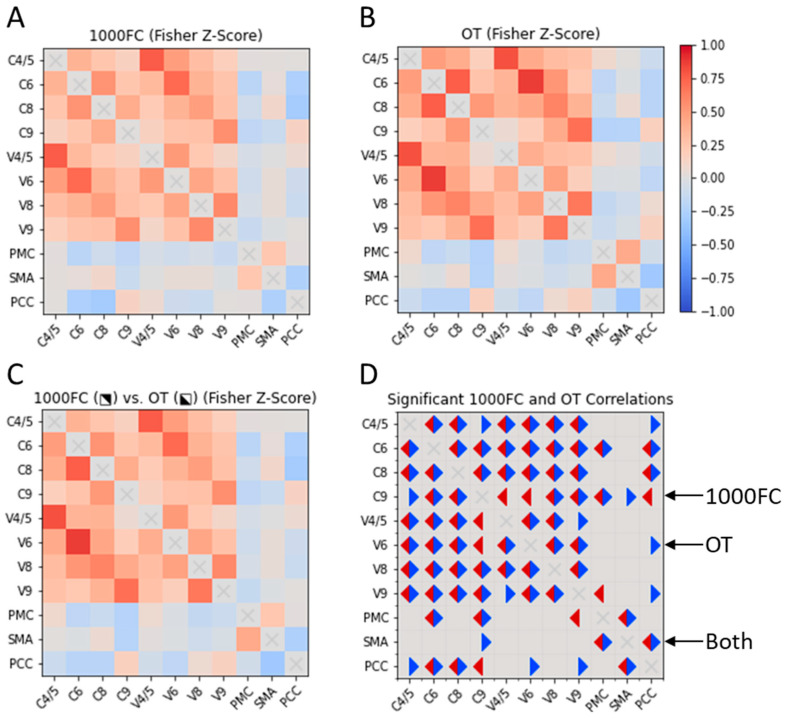
Cross correlation matrices of CON elements in OT individuals and the 1000FC group. Two cross correlation matrices, one for 1000FC (**B**) and the other for the OT dataset (**A**). The OT and 1000FC datasets are also displayed together (**C**) with 1000FC in the upper right portion of the matrix and OT in the bottom left. A final correlation matrix (**D**) represents significant correlations for both datasets. Significant correlations in the OT dataset are indicated with blue triangles and 1000FC are in red. 1000FC = 1000 Functional Connectomes. OT = orthostatic tremor. C = cerebellar lobule. V = cerebellar vermis. PMC = primary motor cortex. SMA = supplementary motor area. PCC = posterior cingulate cortex.

We qualitatively compared RSFC between CON ROIs in the OT and bootstrapped 1000FC datasets. We observed few between-group differences in the datasets (Figure 2). For the OT sample, we observed unique positive intracerebellar correlations. These OT-specific correlations involved C4/5-C9 and V4/5-V9. Within the 1000FC sample, there were positive intracerebellar correlations between C9 and two vermis areas that were not significant within the OT sample. Two sample-specific correlations were identified between putative CON components. The OT-specific correlation was negative RSFC between C9 and SMA. Meanwhile, V9 in the 1000FC group exhibited negative RSFC with the PMC. For further correlation information refer to Figure 2.

#### 3.2.2. Correlations of ROI Pairs on OT Demographics and Disease Variables

**Figure 3:**
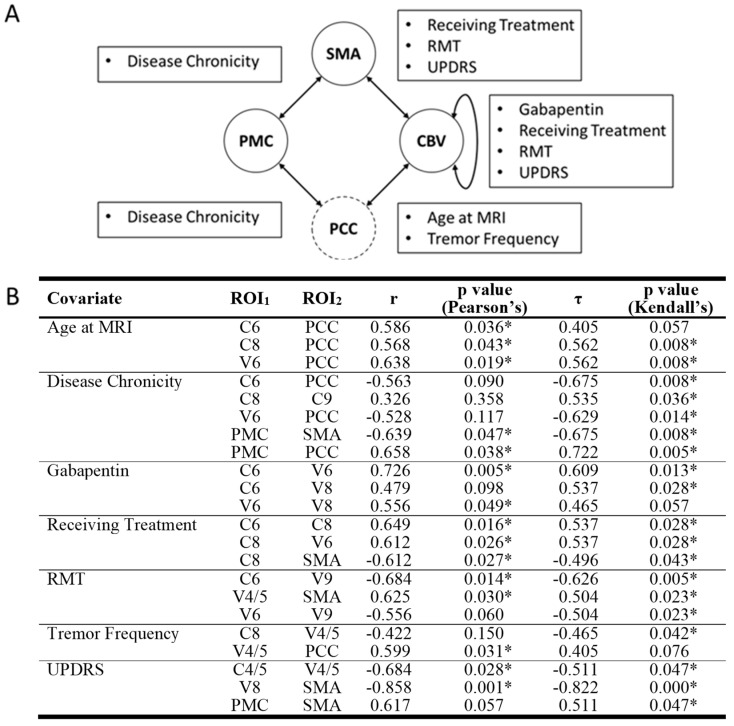
Significant correlations between RSFC of ROI pairs and disease or demographic variables. (**A**) CON components are represented in solid circles while non-CON components are in dashed circles. Arrows between circles signify at least one significant correlation between ROI pairs and disease/demographic variables were detected. Text boxes disclose what disease/demographic variable/s were significant for their associated arrow. (**B**) Both Pearson’s and Kendall’s correlation values and significance values are also displayed. Nonsignificant correlations are italicized. RSFC = resting state functional connectivity. ROI = region of interest. C = cerebellar lobule. CBV = cerebellum and cerebellar vermis. PCC = posterior cingulate cortex. PMC = primary motor cortex. SMA = supplementary motor area. V = cerebellar vermis. * = significant correlation.

Our analysis identified one statistically significant correlation between ROI-ROI RSFC values and demographic or disease variables in the OT group that survived multiple comparisons (Figure 3). Specifically, UPDRS score was a significant negative predictor of RSFC between SMA and V8 (*r* = −0.858, Pearson’s *p* = 0.001, *τ* = −0.822, Kendall’s *p* < 0.001).

While other correlations were not statistically significant after correction for multiple comparisons, several other ROI-ROI RSFC values and demographic/disease variable associations were found that could inform future studies. These include the following. UPDRS was negatively associated with RSFC between SMA and V8 and between C4/5 and V4/5, resting motor threshold was negatively associated with RSFC between C6 and V9 and positively associated with RSFC between SMA and V4/5. All statistically significant correlations had Pearson’s correlation coefficients greater than 0.5 representing large effect sizes. For a comprehensive list of correlations, please see Figure 3.

We also assessed the same associations between CON components and PCC. Regarding disease variables, but none of these associations were found to be statistically significant after correction for multiple comparisons.

### 3.3. Whole-Brain Resting-State Functional Connectivity of Putative CON Components

We also evaluated whole-brain RSFC of CON components using a seed-based approach to measure RSFC and to qualitatively contrast findings from the OT and 1000FC groups. For calculating spatial extent of clusters in the 1000FC sample, the whole dataset was utilized (i.e., not bootstrapped).

#### 3.3.1. RSFC of the PMC

In the OT group, PMC exhibited a pattern of RSFC that included typical motor regions and CON elements (Figure S1, Table S2). Four clusters were present in cerebellum, two positive and two negative. Comparing RSFC of the PMC in the OT group with the same seed region in the 1000FC group revealed a strong resemblance. The spatial correlation of the RSFC patterns was r = 0.82, and spatial overlap between clusters was high (Dice’s coefficient = 0.54). Group differences present could be attributable to the disease process or other between-group differences.

#### 3.3.2. RSFC of the SMA

RSFC of the SMA in OT included many motor regions (Figure S2 and Table S2). RSFC clusters were observed in the bilateral pre- and post-central gyri as well as cerebellum. Clusters were also identified in cingulate gyrus and bilateral insula. Inspection of cluster maps indicated strong similarity between the OT and 1000FC groups (r = 0.81, Dice’s coefficient = 0.31).

#### 3.3.3. RSFC of the Cerebellum

Cerebellar seeds in the OT group had significant positive intracerebellar RSFC as well as significant RSFC with motor regions. Negative clusters were present in the superior frontal gyrus and SMA for most cerebellar seeds. Additional cluster information is in Supplementary Tables S4–S16. Contrasting the RSFC patterns with the 1000FC dataset indicated relatively high spatial correlation (range, r = 0.48–0.73). There was less spatial overlap of clusters than the seed regions described above (Dice’s coefficient = 0.0–0.11). The discrepancy between strong spatial correlation and weaker spatial overlap of clusters is likely due to the difference in sample size between groups. Correlation information and spatial overlap values for individual seeds can be found in Supplementary Table S17.

#### 3.3.4. Small-Volume ROIs

##### RSFC of the PT

The RSFC pattern of the PT in OT contained two clusters (Table S18). The first was the seed cluster, which extended to the left cerebellar cortex. The remaining cluster was negative and in the right superior parietal lobule.

##### RSFC of the VIM

The exhibited RSFC patterns of the left and right VIM in OT were limited to the thalamus near the seed region. For additional cluster information refer to Supplementary Table S18.

##### Spatial Similarity with Normative RSFC: PT and VIM

Comparison between the OT and 1000FC datasets resulted in similar values for all three of these small ROIs. RSFC profiles from the PT, right VIM, and left VIM produced smaller values for spatial correlation and overlap than other seeds. Spatial correlation values between left VIM seeds was the highest, with a value of 0.43 followed by the PT with 0.27 and right VIM with 0.17. No spatial overlap of clusters was observed for the small volume ROIs.

#### 3.3.5. Resting-State Functional Connectivity of a Non-Motor Network (DMN)

PCC was included to assess the integrity of a brain network not typically associated with motor functions—the DMN. Similar to prior reports, the analysis of PCC RSFC revealed several clusters within the CON [51]. We observed RSFC between PCC and PMC, SMA, and cerebellum (Figure S3 and Table S19). Analysis of the 1000FC data alongside OT revealed high spatial correlation and moderate overlap (r = 0.84, Dice’s coefficient = 0.38). Congruence of PCC RSFC between datasets suggests that the DMN is generally maintained in OT, and changes may be limited to CON regions.

## 4. Discussion

Our study used rs-fMRI to measure the RSFC of the putative CON. We observed correlations between OT-related disease variables and CON connectivity. We also observed qualitative differences in ROI-ROI correlations between OT and normal individuals. Robust RSFC within the CON in the OT group was evident and broadly consistent with the comparison group. However, several qualitative differences in RSFC were identified and may potentially be attributable to OT.

### 4.1. Central Oscillatory Network

Our observations of RSFC in OT broadly align with previous studies suggesting that OT may be attributable to cerebellar dysfunction. One prior study reported abnormal connectivity within the DMN and decreased RSFC in the cerebellum and sensorimotor networks [40]. Another reported increased connectivity between the SMA and the lateral cerebellum [6]. We similarly observed qualitative differences between groups in CON RSFC, but our observations did not precisely replicate those reported in prior work. Differences between our findings and prior work may be attributable to methodological differences of RSFC analysis [6].

Research on brain correlates of OT suggests the presence of a CON involving PMC, SMA, cerebellum, PT, and bilateral VIM [10,18]. Although not identified as a “canonical” intrinsic functional brain network, our analysis revealed robust connectivity between CON regions similar to prior studies [11,52,53]. Further, the 1000FC group provided insight regarding normative functional organization of the CON: positive RSFC between PMC and SMA and negative between PMC and cerebellum. We observed similar clusters of RSFC and Dice’s coefficient values across both groups. Despite these similarities, only the OT group had significant RSFC between SMA and cerebellum.

We observed qualitative between-group differences in RSFC between cerebellum and SMA in our analysis. While the 1000FC group’s PMC had more significant RSFC with cerebellar ROIs, in the OT group, cerebellar ROIs exhibited greater RSFC with the SMA. Whether differences in cerebellar RSFC with brain regions emerge in proportion to OT-related disease variables would require longitudinal study of OT.

### 4.2. Functional Connectivity in OT beyond Motor Regions

We also studied the DMN operationalized as the PCC. Our analysis indicated the OT group exhibited more PCC associations than the 1000FC sample. Despite changes between the PCC and CON, RSFC of the PCC was consistent with the expected spatial pattern, suggesting the DMN may be relatively preserved in OT. Analysis also revealed different connectivity of the cerebellum between the groups. This along with the loss of cerebellar RSFC with other CON elements could indicate brain changes in OT may be concentrated in the cerebellum, consistent with OT being associated with cerebellar dysfunction, but additional research would be necessary to comprehensively test this claim [6].

### 4.3. Network Integrity

We observed network resemblance between the OT and 1000FC datasets. Specifically, the spatial correlation of regions between groups was above r = 0.65 for all seeds except the VIM, PT, and certain cerebellar ROIs. Network structure between datasets did not vary to the degree other studies have reported, but we did note reduced RSFC between the cerebellum and other CON components. Differences from prior reports could be attributable to differences in data collection/analysis, small sample sizes, or other factors. Nonetheless, cerebellum has been frequently implicated in OT [3,6,40,51].

### 4.4. Limitations

The age difference of the participants in the 1000FC and OT datasets could have contributed to some findings. Future studies might include age-matched healthy comparison participants to isolate factors related to OT. Although this was not feasible for our study, others have used this approach [6,40,54]. However, strong spatial similarity between our groups for many ROIs suggests that age-related differences were not a major influence. Also, while unavailable for this study, measures such as the Orthostatic Tremor Severity and Disability Scale are now accessible and provide more targeted assessment of OT associated pathology [55]. An additional limitation was the number of individuals in the OT group. Still, our sample size was comparable to other studies of OT [6,40], all of which must contend with the rarity of this complex disease. However, sample size may have contributed to the small number of significant clusters of RSFC for seeds in the OT sample compared to 1000FC. Additionally, small ROIs presented some challenges relative to other ROIs as the VIM and PT are subject to susceptibility artifacts during MRI data collection. Finally, the application of measures of unsteadiness could be particularly informative as a measure of OT severity; unfortunately, such data were not available for this project.

## 5. Conclusions

The findings of our study are consistent with the perspective that the cerebellum may be particularly impacted by OT, and they are consistent with the perspective that further investigation of OT on cerebellar RSFC may be warranted. We also observed novel associations between demographic/disease variables and RSFC of ROI pairs. Overall, our analysis indicated that the integrity of the brain’s intrinsic functional networks appeared to be broadly maintained in OT, suggesting that functional brain changes in OT may be regionally specific and functionally selective processes. In the future, functional brain changes could potentially serve as biomarkers for diagnosis, monitoring, and treatment of OT, motivating further research of this rare and complex movement disorder.

## Figures and Tables

**Table 1:** Participant Characteristics.

Age at MRI	Sex	Age of Onset	Age at Dx	Tr. Freq. (Hz)	RMT	Tx	Family History	UPDRS
70	F	67	70	12	32	G	No	23
78	F	75	75	13	*	—	No	27
68	F	60	64	14	25	G	No	*
76	F	55	59	14	52	—	Yes	8
61	F	46	61	15	33	G	No	7
60	F	45	52	15	43	C	No	19
78	F	67	70	15	33	—	No	4
70	F	64	66	14	28	C	No	16
41	M	40	40	14	51	G + C	No	14
79	F	49	52	16	54	G + C	No	10
62	F	47	47	15	60	C	No	*
76	F	57	58	14	48	C	No	28
67	F	30	30	14	10	C	Yes	*

Participant information including age at MRI, sex, age of OT diagnosis, age of OT onset, other diagnosed movement disorders, RMT, tremor frequency, current OT treatments, if there is a family history of movement disorders, and UPDRS score. * = data unavailable. C = clonazepam. Dx = diagnosis. Freq = frequency. G = gabapentin. G + C = gabapentin and clonazepam. RMT = resting motor threshold. Tr = tremor. Tx = treatment.

**Table 2:** RSFC seed information. Seed information including source publication and MNI coordinates used for generation of each seed.

		Source Coordinates (MNI)		
ROI	Source	X	Y	Z
Pontine Tegmentum	Schöberl et al., 2017 [19]	+5.0	−38.0	−42.0
Ventral intermediate Nucleus (Thalamus)	Yamada et al., 2010 [41]	+/−11.5	−15.5	−1.0
Thumb	Roux et al., 2018 [42]	+46.6	−22.8	+56.2
Supplementary Motor Area	Gallea et al., 2016 [6]	−9.0	+8.0	+56.0
Posterior Cingulate Cortex	Andrews-Hanna et al., 2010 [43]	−8.0	−56.0	+26.0

## Data Availability

Data used in the present study from the 1000FC dataset are available from the International Neuroimaging Data-Sharing Initiative [37]. Data on individuals with OT are available upon request to the corresponding author. The data are not publicly available due to privacy concerns.

## Supplementary Materials

The following supporting information can be downloaded at: https://www.mdpi.com/article/10.3390/brainsci14030219/s1, Figure S1: Significant clusters from RSFC analysis of PMC in OT and 1000FC, Figure S2: Significant clusters from RSFC analysis of SMA in OT and 1000FC, Figure S3: Significant clusters from RSFC analysis of PCC in OT and 1000FC, Table S1: Previously Reported Brain Activity and Demographic or Tremor Variables, Table S2: Resting-State Functional Connectivity Results for PMC, Table S3: Resting-State Functional Connectivity Results for SMA, Table S4: Resting-State Functional Connectivity for Vermis 9, Table S5: Resting-State Functional Connectivity for Vermis 8, Table S6: Resting-State Functional Connectivity for Vermis 7, Table S7: Resting-State Functional Connectivity for Vermis 6, Table S8: Resting-State Functional Connectivity for Vermis 4/5, Table S9: Resting-State Functional Connectivity for Cerebellum 9 R, Table S10: Resting-State Functional Connectivity for Cerebellum 9 L, Table S11: Resting-State Functional Connectivity for Cerebellum 8 R, Table S12: Resting-State Functional Connectivity for Cerebellum 8 L, Table S13: Resting-State Functional Connectivity for Cerebellum 6 R, Table S14: Resting-State Functional Connectivity for Cerebellum 6 L, Table S15: Resting-State Functional Connectivity for Cerebellum 4/5 R, Table S16: Resting-State Functional Connectivity for Cerebellum 4/5 L, Table S17: Correlations and Dice’s Coefficients Between Datasets for Cerebellar ROIs, Table S18: Resting-State Functional Connectivity Results for Small Volume ROIs, Table S19: Resting-State Functional Connectivity Results for PCC.
