# Supplementary material for: Measurement of Functional Brain Network Connectivity in People with Orthostatic Tremor
Source: Brain Sci. 2024 Feb 27;14(3):219. doi: 10.3390/brainsci14030219 (PMC10968606; doi:10.3390/brainsci14030219)
Supplement: Supplementary file 1 [file brainsci-14-00219-s001.zip › Manuscript Figures-Supp.pdf]

Figure S1

Significant clusters from RSFC analysis of PMC in OT and 1000FC

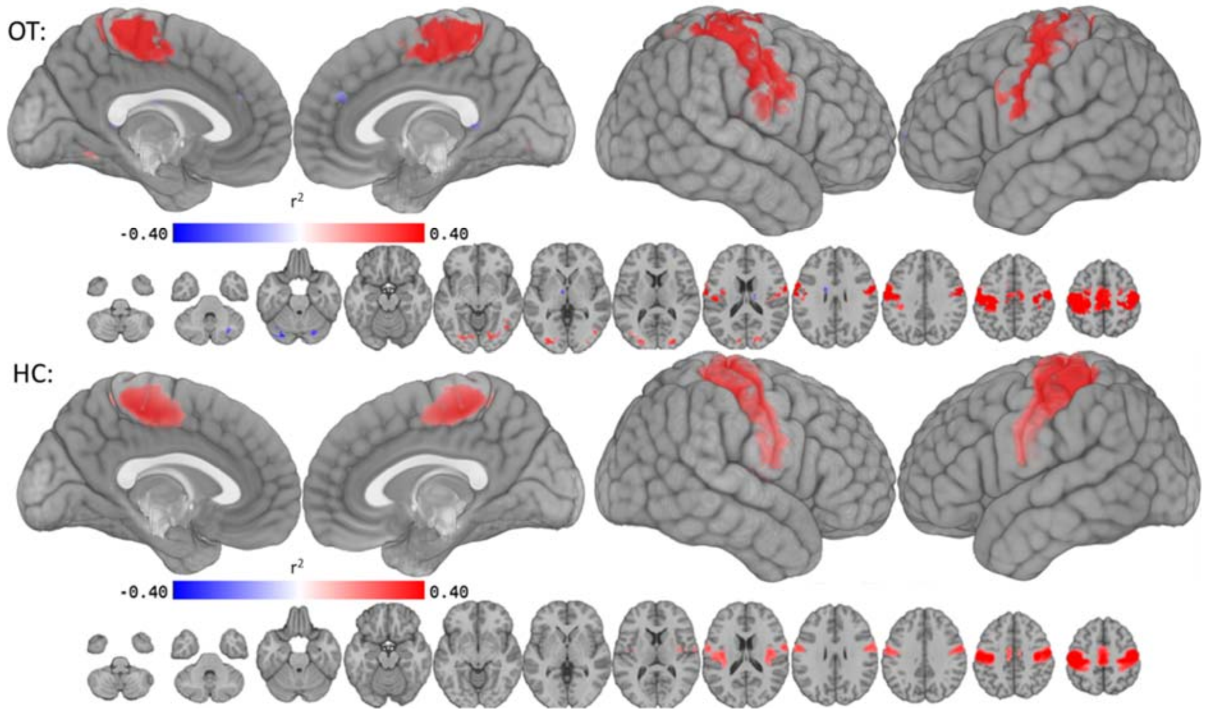

Figure S1: Resting state functional connectivity with primary motor cortex in (A,C) patients with orthostatic tremor and (B,D) healthy young adults. Overall PMC RSFC with GSR for the OT and HC samples depicted on the MNI-152 template brain in radiological view. Three-dimensional renderings showing RSFC patterns with the PMC (perspectives, left to right and top to bottom: left medial, right medial, right lateral, left lateral).

Figure S2

Significant clusters from RSFC analysis of SMA in OT and 1000FC

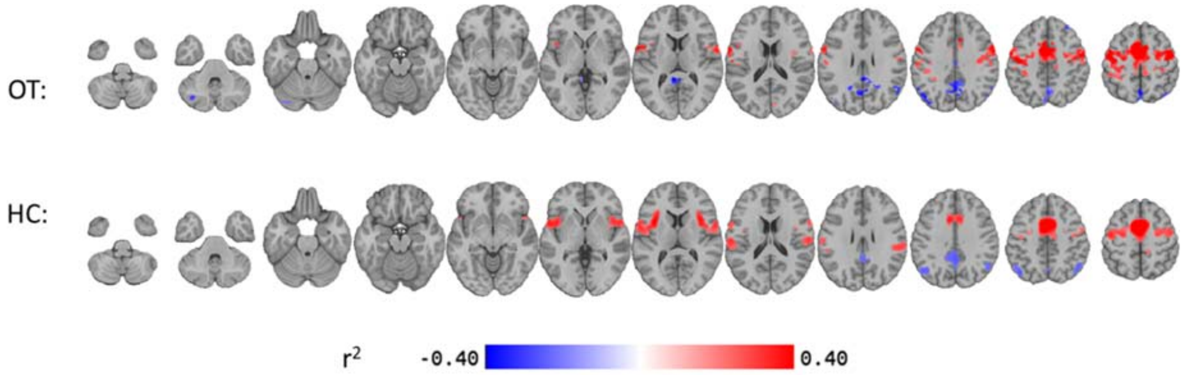

Figure S2: Resting state functional connectivity with the supplementary motor area in orthostatic tremor and healthy young adults. Overall RSFC of the SMA with GSR for the OT and HC samples depicted on the MNI-152 template brain in radiological view. Smaller clusters are circled for ease of viewing.

Figure S3

Significant clusters from RSFC analysis of PCC in OT and 1000FC

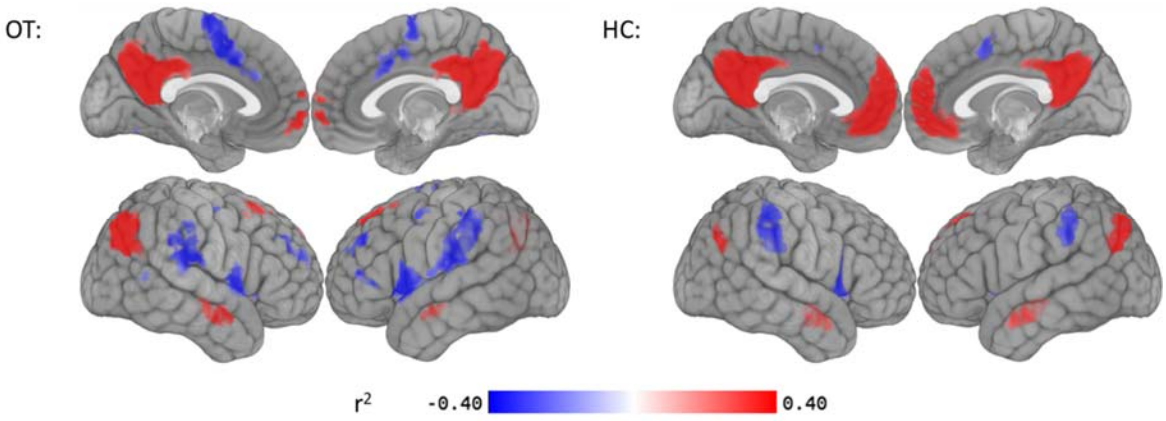

Figure S3: Resting state functional connectivity with posterior cingulate cortex is maintained in orthostatic tremor. Overall PCC RSFC with GSR for the OT and HC samples depicted on the MNI-152 template brain in radiological view. Three-dimensional renderings showing RSFC patterns with the CBV (perspectives, left to right and top to bottom: right medial, left medial, right lateral, left lateral).
