# Supplementary material for: Measurement of Functional Brain Network Connectivity in People with Orthostatic Tremor
Source: Brain Sci. 2024 Feb 27;14(3):219. doi: 10.3390/brainsci14030219 (PMC10968606; doi:10.3390/brainsci14030219)
Supplement: Supplementary file 1 [file brainsci-14-00219-s001.zip › Manuscript Tables-Supp.pdf]

Table S1

Previously Reported Brain Activity and Demographic or Tremor Variables

Source	Demographic or Tremor Variable	CON Region
Benito-León	Disease Duration	DMN
Gallea	Disease Duration	C6
		C9
		SMA
		V6
		V9
	EMG	C4-5
		SMA
		V8
	Tremor Severity	C4-5
		C6
		C9
		SMA
		V4-5

Table S1. Prior reported variable and ROI-ROI RSFC associations from Gallea and colleagues and Benito-León and colleagues. C = Cerebellar lobule. CON = Central oscillatory network. DMN = Default mode network, EMG = Electromyography. V = Cerebellar vermis.

Table S2

Resting-State Functional Connectivity Results for PMC

				MNI Coordinates			
**#**	**k**	**T**	**v**	**x**	**y**	**z**	**Region(s)**
**1**	10270	16.003	+	-39	-25	59	Bilateral precentral gyrus
							Bilateral postcentral gyrus
							Bilateral paracentral lobule
							Bilateral superior parietal lobule
							Bilateral superior frontal gyrus
							Bilateral supramarginal gyrus
							Bilateral middle frontal gyrus (caudal)
							Right posterior cingulate
							Right precuneus
**2**	166	11.432	+	25	-89	11	Right lateral occipital gyrus
**3**	165	9.579	+	-25	-89	9	Left lateral occipital gyrus
							Left inferior parietal lobule
**4**	121	11.493	+	-49	-19	19	Left postcentral gyrus
							Left supramarginal gyrus
							Left insula
**5**	119	6.998	+	9	-75	-5	Right lingual gyrus
**6**	89	7.204	+	-33	-79	-11	No GM
**7**	77	6.446	-	-33	-71	-23	Left cerebellar cortex
							Left fusiform gyrus
**8**	65	12.134	+	53	-11	19	Right postcentral gyrus
							Right insula
							Right supramarginal gyrus

**9**	56	10.304	-	23	61	7	Right middle frontal gyrus (rostral)
**10**	52	6.171	-	23	-73	-25	Right cerebellar cortex
**11**	46	6.38	+	-39	-9	19	Left insula
							Left precentral gyrus
							Left postcentral gyrus
**12**	45	8.071	+	45	-53	-13	Right fusiform gyrus
							Right inferior temporal gyrus
**13**	38	9.56	-	-3	-43	7	No GM
**14**	34	5.353	+	47	-75	7	Right lateral occipital gyrus
**15**	33	6.33	-	33	-67	-37	Right cerebellar cortex
**16**	27	6.618	-	15	-13	23	Right thalamus proper
							Right caudate
**17**	26	6.964	+	-35	-77	7	No GM
**18**	26	6.636	+	-53	-19	27	No GM
**19**	25	5.759	+	43	-65	-9	Left superior frontal gyrus
							Right caudal anterior cingulate
**20**	25	7.629	-	-3	43	25	Right lateral occipital gyrus
**21**	21	6.892	+	19	-57	-11	No GM
**22**	21	6.339	+	-13	-89	15	Right lingual gyrus
							Right cerebellar cortex

Table S2. Clusters of significant RSFC for the PMC including coordinates and structures of the brain each cluster is present in. RSFC = resting state functional connectivity. PMC = primary motor cortex. k = cluster size in voxels. T = peak T statistic in the cluster. V = positive or negative nature of cluster to seed.

Table S3

Resting-State Functional Connectivity Results for SMA

				MNI Coordinates			
**#**	**k**	**T**	**v**	**x**	**y**	**z**	**Region(s)**
**1**	8978	23.034	+	9	-9	59	Bilateral precentral gyrus
							Bilateral superior frontal gyrus
							Bilateral paracentral lobule
							Bilateral postcentral gyrus
							Left superior parietal lobule
							Bilateral middle frontal gyrus (caudal)
							Bilateral posterior cingulate
							Bilateral caudal anterior cingulate
							Bilateral supramarginal gyrus
**2**	854	7.316	-	7	-71	41	Bilateral precuneus
							Bilateral cingulate gyrus (isthmus)
							Bilateral superior parietal lobule
**3**	217	8.045	+	61	-5	13	Right precentral gyrus
							Right postcentral gyrus
							Right superior temporal gyrus
**4**	153	8.915	-	-41	-83	33	Left inferior parietal lobule
							Left supramarginal gyrus
**5**	151	8.088	-	-7	-43	1	Left cingulate gyrus (isthmus)
							Left precuneus
**6**	101	6.788	-	-39	-77	-23	Left cerebellar cortex
**7**	98	7.394	+	27	-39	63	Right superior parietal lobule

**8**	92	6.284	-	51	-55	25	Right inferior parietal lobule
**9**	55	7.131	+	39	-1	17	Right insula
**10**	53	7.173	+	45	-31	41	Right supramarginal gyrus
**11**	48	6.884	+	-47	13	1	Left pars opercularis
							Left insula
**12**	43	7.619	+	-39	-3	13	Left insula
							Left precentral gyrus
							Left postcentral gyrus
**13**	41	8.695	+	21	-51	65	Right superior parietal lobule
**14**	35	7.597	+	7	-45	71	Right superior parietal lobule
							Right precuneus
**15**	34	6.746	-	29	35	49	Right middle frontal gyrus (caudal)
							Right middle frontal gyrus (rostral)
**16**	33	5.566	-	9	-41	7	Bilateral cingulate gyrus (isthmus)
**17**	27	5.836	-	41	-69	53	Right inferior parietal lobule
**18**	26	5.814	+	-49	9	21	Left precentral gyrus
**19**	25	5.718	-	1	-19	35	Bilateral posterior cingulate
**20**	23	6.307	-	25	-57	29	No GM
**21**	20	5.864	+	-35	7	23	Left middle temporal gyrus
**22**	20	5.057	-	-63	-17	-13	No GM
**23**	19	9.321	+	7	-85	19	Left superior parietal lobule
**24**	19	7.018	+	-35	-55	63	Right cuneus

Table S3. Clusters of significant RSFC for SMA including coordinates and structures of the brain each cluster is present in. RSFC = resting state functional connectivity. SMA = supplementary motor area. k = cluster size in voxels. T = peak T statistic in the cluster. V = positive or negative nature of cluster to seed.

Table S4

Resting-State Functional Connectivity for Vermis 9

				MNI Coordinates			
**#**	**k**	**T**	**v**	**x**	**y**	**z**	**Region(s)**
**1**	710	17.802	+	-1	-53	-31	Bilateral cerebellar cortex
**2**	84	7.493	+	-33	-63	-39	Left cerebellar cortex
**3**	35	8.034	+	-21	-65	-25	Left cerebellar cortex
**4**	33	7.384	-	-53	11	29	Left precentral gyrus
							Left pars opercularis
**5**	24	7.774	+	-33	-61	-49	Left cerebellar cortex
**6**	18	5.915	-	43	-65	-21	No GM
**7**	18	7.778	-	19	43	1	Right cerebellar cortex
							Right fusiform gyrus

Table S4. Clusters of significant RSFC for cerebellar vermis 9 including coordinates and structures of the brain each cluster is present in. RSFC = resting state functional connectivity. k = cluster size in voxels. T = peak T statistic in the cluster. V = positive or negative nature of cluster to seed.

Table S5

Resting-State Functional Connectivity for Vermis 8

				**MNI Coordinates**			
**#**	**k**	**T**	**v**	**x**	**y**	**z**	**Region(s)**
**1**	1590	13.958	+	3	-63	-37	Bilateral cerebellar cortex
**2**	50	7.099	+	3	-65	-17	Bilateral cerebellar cortex
**3**	41	6.418	+	39	-59	-37	Right cerebellar cortex
**4**	32	8.312	-	19	43	-3	No GM
**5**	24	5.85	-	15	-55	61	Right superior parietal lobule
**6**	18	5.154	-	11	41	1	Right rostral anterior cingulate gyrus
							Right medial orbitofrontal cortex

Table S5. Clusters of significant RSFC for cerebellar vermis 8 including coordinates and structures of the brain each cluster is present in. RSFC = resting state functional connectivity. k = cluster size in voxels. T = peak T statistic in the cluster. V = positive or negative nature of cluster to seed.

Table S6

Resting-State Functional Connectivity for Vermis 7

				MNI Coordinates			
**#**	**k**	**T**	**v**	**x**	**y**	**z**	**Region(s)**
**1**	1318	13.334	+	3	-77	-27	Bilateral cerebellar cortex
			+				Left lingual gyrus
**2**	179	7.731	-	11	43	-3	Bilateral rostral anterior cingulate gyrus
							Bilateral medial orbitofrontal cortex
							Bilateral superior frontal gyrus
**3**	105	8.452	-	35	-33	61	Right postcentral gyrus
							Right superior parietal lobule
**4**	82	9.291	-	-11	55	7	Left superior frontal gyrus
**5**	54	7.395	-	7	55	21	Bilateral superior frontal gyrus
**6**	45	8.034	-	-19	-13	73	Left precentral gyrus
							Left superior frontal gyrus
**7**	38	6.469	-	45	-17	55	Right postcentral gyrus
							Right precentral gyrus
**8**	26	5.315	-	-63	-11	33	Left postcentral gyrus
							Left precentral gyrus
**9**	25	6.01	-	-5	39	7	Left rostral anterior cingulate gyrus
**10**	24	5.652	-	-17	45	-3	No GM
**11**	23	6.138	-	15	65	9	Right superior frontal gyrus
**12**	21	6.24	-	15	-41	63	Right precuneus
**13**	18	6.094	-	-39	-51	17	No GM

Table S6. Clusters of significant RSFC for cerebellar vermis 7 including coordinates and structures of the brain each cluster is present in. RSFC = resting state functional connectivity. k = cluster size in voxels. T = peak T statistic in the cluster. V = positive or negative nature of cluster to seed.

Table S7

Resting-State Functional Connectivity for Vermis 6

				MNI Coordinates			
**#**	**k**	**T**	**v**	**x**	**y**	**z**	**Region(s)**
**1**	2021	17.514	+	5	-57	-19	Bilateral cerebellar cortex
			+				Bilateral lingual gyrus
			+				Bilateral fusiform gyrus
**2**	243	8.728	-	3	49	-1	Bilateral medial orbitofrontal cortex
							Bilateral superior frontal gyrus
							Bilateral rostral anterior cingulate gyrus
**3**	120	7.059	+	-5	-73	-37	Left cerebellar cortex
**4**	63	7.131	-	9	55	41	Right superior frontal gyrus
**5**	46	5.86	-	-3	41	39	Bilateral superior frontal gyrus
**6**	30	8.411	-	-55	-35	3	Left superior temporal sulcus (bank)
**7**	29	6.813	-	-55	31	15	Left pars opercularis
							Left pars triangularis
**8**	29	7.436	-	-27	29	57	Left superior frontal gyrus
							Left middle frontal gyrus (caudal)
**9**	28	6.667	-	3	39	47	Bilateral superior frontal gyrus
**10**	27	6.146	+	-31	-57	-45	Left cerebellar cortex
**11**	21	6.23	+	29	-67	9	Right superior frontal gyrus
							Right rostral anterior cingulate gyrus

**12**	21	6.436	-	7	51	13	No GM
**13**	18	6.831	+	21	-47	-17	Right cerebellar cortex

Table S7. Clusters of significant RSFC for cerebellar vermis 6 including coordinates and structures of the brain each cluster is present in. RSFC = resting state functional connectivity. k = cluster size in voxels. T = peak T statistic in the cluster. V = positive or negative nature of cluster to seed.

Table S8

Resting-State Functional Connectivity for Vermis 4/5

				**MNI Coordinates**			
**#**	**k**	**T**	**v**	**x**	**y**	**z**	**Region(s)**
**1**	1835	15.52	+	1	-51	-13	Bilateral cerebellar cortex
							Bilateral lingual gyrus
							Bilateral cingulate gyrus (isthmus)
							Left parahippocampal gyrus
							Left thalamus proper
							Bilateral precuneus
							Left fusiform gyrus
**2**	61	6.022	+	-35	-57	-43	Left cerebellar cortex
**3**	52	6.324	+	25	-57	-47	Right cerebellar cortex
**4**	47	6.673	-	-1	-65	45	Bilateral precuneus
**5**	45	8.492	+	-33	-53	9	No GM
**6**	31	7.768	-	9	9	9	Right caudate
							Right thalamus proper
**7**	22	6.978	+	7	-15	75	Left caudate
							Left nucleus accumbens
**8**	22	5.879	-	-3	7	5	Right paracentral lobule
							Right precentral gyrus
**9**	21	7.31	-	-3	51	35	Bilateral superior frontal gyrus
**10**	18	6.708	+	-23	-55	-47	Left cerebellar cortex
**11**	18	6.422	-	-11	17	-1	Left caudate
							Left putamen

Table S8. Clusters of significant RSFC for cerebellar vermis 4/5 including coordinates and structures of the brain each cluster is present in. RSFC = resting state functional connectivity. k =

cluster size in voxels. T = peak T statistic in the cluster. V = positive or negative nature of cluster to seed.

Table S9

Resting-State Functional Connectivity for Cerebellum 9 R

#	k	T	v	MNI Coordinates x	y	z	Region(s)
**1**	1140	16.691	+	5	-55	-49	Bilateral cerebellar cortex
**2**	210	8.412	+	39	-61	-39	Right cerebellar cortex
**3**	58	6.994	-	-27	-85	23	Left inferior parietal lobule
							Left superior parietal lobule
**4**	57	7.067	+	57	-57	29	Right inferior parietal lobule
**5**	51	7.36	-	53	25	3	Right pars opercularis
							Right pars triangularis
**6**	39	6.46	+	-17	-77	-43	Left cerebellar cortex
**7**	33	5.781	-	1	-11	53	Bilateral superior frontal gyrus
							Bilateral paracentral lobule
**8**	29	8.175	-	-57	5	41	Left precentral gyrus
							Left middle frontal gyrus (caudal)
**9**	28	8.012	+	-27	-67	-37	Left cerebellar cortex
**10**	27	6.396	+	-33	-65	-31	Left cerebellar cortex
**11**	27	6.927	+	-5	-47	5	Left cingulate gyrus (isthmus)
**12**	27	7.605	+	11	-39	31	Right precuneus
			+				Right cingulate gyrus (isthmus)
**13**	27	5.776	-	45	39	27	Right middle frontal gyrus (rostral)
**14**	26	5.425	+	-1	-47	19	Left cingulate gyrus (isthmus)
**15**	24	7.974	-	5	21	39	Bilateral superior frontal gyrus

							Right caudal anterior cingulate
**16**	21	6.646	-	33	23	5	Left precentral gyrus
**17**	21	5.588	-	57	-37	9	Right insula
							Right pars opercularis
**18**	21	6.388	-	-53	9	29	Right superior temporal sulcus (bank)
**19**	20	6.048	-	-35	-53	59	Left superior parietal lobule
**20**	18	6.637	-	43	-57	-1	No GM

Table S9. Clusters of significant RSFC for right cerebellar lobule 6 including coordinates and structures of the brain each cluster is present in. RSFC = resting state functional connectivity. k = cluster size in voxels. T = peak T statistic in the cluster. V = positive or negative nature of cluster to seed.

Table S10

Resting-State Functional Connectivity for Cerebellum 9 L

				MNI Coordinates			
**#**	**k**	**T**	**v**	**x**	**y**	**z**	**Region(s)**
**1**	614	10	+	-7	-59	-51	Bilateral cerebellar cortex
**2**	96	7.537	+	-1	-59	-29	Bilateral cerebellar cortex
**3**	75	7.556	+	-31	-63	-47	Left cerebellar cortex
**4**	39	6.87	+	-31	-65	-31	Left cerebellar cortex
**5**	20	6.267	+	13	-43	-49	Right cerebellar cortex
**6**	18	7.136	-	-5	9	13	Left caudate

Table S10. Clusters of significant RSFC for left cerebellar lobule 9 including coordinates and structures of the brain each cluster is present in. RSFC = resting state functional connectivity. k = cluster size in voxels. T = peak T statistic in the cluster. V = positive or negative nature of cluster to seed.

Table S11

Resting-State Functional Connectivity for Cerebellum 8 R

				**MNI Coordinates**			
**#**	**k**	**T**	**v**	**x**	**y**	**z**	**Region(s)**
**1**	2156	12.825	+	25	-59	-33	Bilateral cerebellar cortex
							Right fusiform gyrus
							Right lingual gyrus
**2**	161	6.352	+	-21	-51	-55	Left cerebellar cortex
**3**	120	6.877	-	1	-61	31	Bilateral precuneus
**4**	40	6.275	-	23	31	43	No GM
**5**	39	6.781	+	-43	-13	-17	Left superior temporal gyrus
**6**	37	8.075	+	-67	-33	29	Left supramarginal gyrus
**7**	31	5.878	-	-59	-11	15	Left postcentral gyrus
**8**	30	5.984	-	7	49	11	Right rostral anterior cingulate gyrus
							Right superior frontal gyrus
**9**	23	7.495	+	67	-25	37	Right supramarginal gyrus

Table S11. Clusters of significant RSFC for right cerebellar lobule 8 including coordinates and structures of the brain each cluster is present in. RSFC = resting state functional connectivity. k = cluster size in voxels. T = peak T statistic in the cluster. V = positive or negative nature of cluster to seed.

Table S12

Resting-State Functional Connectivity for Cerebellum 8 L

				MNI Coordinates			
**#**	**k**	**T**	**v**	**x**	**y**	**z**	**Region(s)**
**1**	1927	11.421	+	-11	-71	-47	Bilateral cerebellar cortex
							Left lingual gyrus
**2**	150	8.389	+	57	-33	33	Right supramarginal gyrus
							Right superior temporal gyrus
**3**	79	6.922	-	-63	-5	-21	Left middle temporal gyrus
							Left superior temporal gyrus
**4**	74	6.727	+	9	-69	-43	Right cerebellar cortex
**5**	69	7.321	-	-33	21	53	Left middle frontal gyrus (caudal)
							Left superior frontal gyrus
**6**	68	7.175	-	-9	-65	21	Left precuneus
**7**	45	6.706	-	1	-65	33	Bilateral precuneus
**8**	37	5.88	-	7	69	13	Bilateral superior frontal gyrus
**9**	32	8.863	-	-49	9	-23	Left superior temporal gyrus
							Left middle temporal gyrus
**10**	30	5.149	+	-69	-33	31	Left supramarginal gyrus
							Left superior temporal gyrus
**11**	27	7.121	-	-53	19	29	Left pars opercularis
							Left middle frontal gyrus (rostral)
							Left middle frontal gyrus (caudal)
							Left precentral gyrus
**12**	26	7.912	-	-49	13	43	Left middle frontal gyrus (caudal)

**13**	25	6.618	-	-7	69	7	Left superior frontal gyrus
**14**	25	5.763	-	-3	-61	19	Bilateral precuneus
							Bilateral cingulate gyrus (isthmus)
**15**	22	6.146	+	55	7	-1	Right superior temporal gyrus
							Right precentral gyrus
**16**	21	4.842	+	-37	-45	-35	Left cerebellar cortex
**17**	21	7.98	+	3	-1	73	Left posterior cingulate
							Left precuneus
**18**	21	5.755	-	-7	-37	33	Right superior frontal gyrus
**19**	18	5.682	+	-1	-51	-1	Left cingulate gyrus (isthmus)
							Bilateral cerebellar cortex

Table S12. Clusters of significant RSFC for left cerebellar lobule 8 including coordinates and structures of the brain each cluster is present in. RSFC = resting state functional connectivity. k = cluster size in voxels. T = peak T statistic in the cluster. V = positive or negative nature of cluster to seed.

Table S13

Resting-State Functional Connectivity for Cerebellum 6 R

				MNI Coordinates			
**#**	**k**	**T**	**v**	**x**	**y**	**z**	**Region(s)**
**1**	3868	13.908	+	33	-53	-33	Bilateral cerebellar cortex
							Left lingual gyrus
							Left fusiform gyrus
**2**	118	8.581	-	-63	-9	11	Left postcentral gyrus
							Left precentral gyrus
							Left insula
**3**	90	6.229	-	3	57	-3	Bilateral medial orbitofrontal cortex
							Bilateral superior frontal gyrus
							Bilateral rostral anterior cingulate gyrus
**4**	62	6.774	-	-41	-9	35	No GM
**5**	49	8.839	-	5	-27	49	Right paracentral lobule
**6**	27	7.675	-	-47	-69	-1	Left lateral occipital gyrus
**7**	27	6.314	-	-7	-49	55	Left precuneus
**8**	23	6.663	-	59	-3	33	Right precentral gyrus
**9**	22	6.677	-	47	-17	49	Right postcentral gyrus
**10**	20	6.751	+	-3	-7	9	Bilateral thalamus proper
**11**	18	5.807	-	-57	-19	53	Left postcentral gyrus
							Left precentral gyrus

Table S13. Clusters of significant RSFC for right cerebellar lobule 6 including coordinates and structures of the brain each cluster is present in. RSFC = resting state functional connectivity. k = cluster size in voxels. T = peak T statistic in the cluster. V = positive or negative nature of cluster to seed.

Table S14

Resting-State Functional Connectivity for Cerebellum 6 L

				MNI Coordinates			
**#**	**k**	**T**	**v**	**x**	**y**	**z**	**Region(s)**
**1**	2899	14.489	+	-35	-57	-25	Bilateral cerebellar cortex
			+				Bilateral lingual gyrus
			+				Left fusiform gyrus
**2**	564	7.887	-	11	-51	17	Bilateral precuneus
							Bilateral cingulate gyrus (isthmus)
**3**	140	9.5	+	35	-59	-23	Right cerebellar cortex
**4**	124	6.437	-	3	57	-5	Bilateral medial orbitofrontal cortex
							Bilateral rostral anterior cingulate gyrus
							Left superior frontal gyrus
**5**	84	6.637	-	-23	23	41	Left middle frontal gyrus (caudal)
							Left superior frontal gyrus
**6**	75	7.771	-	-11	49	17	Left inferior parietal lobule
**7**	75	7.377	-	-35	-79	45	Bilateral superior frontal gyrus
**8**	72	6.97	-	-13	-45	41	Left precuneus
**9**	68	8.392	+	63	-31	25	Right supramarginal gyrus
**10**	52	6.444	-	-47	-67	35	Left inferior parietal lobule
**11**	45	7.023	-	-3	41	-9	Left rostral anterior cingulate gyrus
							Left medial orbitofrontal cortex
**12**	44	7.556	-	5	59	31	Right superior frontal gyrus

**13**	37	6.081	-	-3	-43	33	Bilateral precuneus
							Bilateral posterior cingulate
							Left cingulate gyrus (isthmus)
**14**	36	6.849	-	-13	69	5	Left superior frontal gyrus
**15**	33	6.891	-	39	-29	63	Right postcentral gyrus
**16**	31	5.891	+	9	-43	-27	Right cerebellar cortex
**17**	30	7.271	-	-49	9	-23	Left superior temporal gyrus
							Left middle temporal gyrus
**18**	29	7.448	-	-23	45	37	Left postcentral gyrus
**19**	29	5.272	-	-41	-25	53	Left middle frontal gyrus (rostral)
							Left superior frontal gyrus
**20**	28	7.18	+	53	-33	37	Bilateral superior frontal gyrus
**21**	28	6.926	-	3	65	9	No GM
**22**	26	5.91	-	11	-25	61	Bilateral paracentral lobule
**23**	25	5.86	-	39	-19	55	Right precentral gyrus
**24**	23	10.205	+	-5	-47	-3	Left superior temporal sulcus (bank)
**25**	23	6.322	-	-49	-39	1	Bilateral cerebellar cortex
							Left cingulate gyrus (isthmus)
**26**	22	5.838	-	-57	1	-17	Left middle temporal gyrus
							Left superior temporal gyrus

Table S14. Clusters of significant RSFC for left cerebellar lobule 6 including coordinates and structures of the brain each cluster is present in. RSFC = resting state functional connectivity. k = cluster size in voxels. T = peak T statistic in the cluster. V = positive or negative nature of cluster to seed.

Table S15

Resting-State Functional Connectivity for Cerebellum 4/5 R

				MNI Coordinates			
**#**	**k**	**T**	**v**	**x**	**y**	**z**	**Region(s)**
**1**	1759	17.035	+	-5	-53	-23	Bilateral cerebellar cortex
							Bilateral lingual gyrus
							Right fusiform gyrus
							Right parahippocampal gyrus
**2**	203	7.715	-	-9	57	-1	Bilateral superior frontal gyrus
							Bilateral medial orbitofrontal cortex
**3**	59	6.874	-	-49	11	51	Left middle frontal gyrus (caudal)
**4**	54	6.544	-	-51	-51	39	Left supramarginal gyrus
							Left inferior parietal lobule
**5**	46	9.28	-	15	-67	39	No GM
**6**	40	6.368	-	-1	-65	33	Bilateral precuneus
**7**	30	8.145	+	-17	-49	-29	Left cerebellar cortex
**8**	29	5.848	+	-3	-13	59	Bilateral paracentral lobule
							Left superior frontal gyrus
**9**	21	6.616	-	-59	-59	23	Left supramarginal gyrus
							Left inferior parietal lobule
**10**	21	7.148	-	57	-55	31	Right inferior parietal lobule
**11**	20	6.409	+	-5	-13	75	Left precentral gyrus
							Left paracentral lobule
**12**	18	8.289	-	13	25	11	Right caudate

Table S15. Clusters of significant RSFC for right cerebellar lobules 4/5 including coordinates and structures of the brain each cluster is present in. RSFC = resting state functional

connectivity. k = cluster size in voxels. T = peak T statistic in the cluster. V = positive or negative nature of cluster to seed.

Table S16

Resting-State Functional Connectivity for Cerebellum 4/5 L

				MNI Coordinates			
**#**	**k**	**T**	**v**	**x**	**y**	**z**	**Region(s)**
**1**	2031	14.199	+	-13	-41	-17	Bilateral cerebellar cortex
							Bilateral lingual gyrus
							Left parahippocampal gyrus
							Bilateral fusiform gyrus
							Left thalamus proper
							Left cingulate gyrus (isthmus)
**2**	30	5.836	-	11	-77	53	Right superior parietal lobule
**3**	26	5.518	+	-29	-61	-43	Left cerebellar cortex
**4**	26	6.139	-	31	37	-1	No GM
**5**	22	5.948	-	3	49	19	Bilateral superior frontal gyrus
							Right rostral anterior cingulate gyrus
**6**	21	6.994	+	-7	-57	-33	Left pars opercularis
							Left precentral gyrus
**7**	21	6.592	+	-35	-55	11	No GM
**8**	21	7.358	-	-53	15	-3	Left cerebellar cortex
**9**	18	8.781	-	31	27	-15	Right lateral orbitofrontal cortex

Table S16. Clusters of significant RSFC for left cerebellar lobules 4/5 including coordinates and structures of the brain each cluster is present in. RSFC = resting state functional connectivity. k = cluster size in voxels. T = peak T statistic in the cluster. V = positive or negative nature of cluster to seed.

Table S17

Correlations and Dice's Coefficients Between Datasets for Cerebellar ROIs

ROI	Correlation (*r*)	Dice's Coefficient
Cerebellum 4/5 L	0.612027	0.106489
Cerebellum 4/5 R	0.624852	0.028235
Cerebellum 6 L	0.731716	0.053952
Cerebellum 6 R	0.70641	0.009332
Cerebellum 8 L	0.637379	0.02503
Cerebellum 8 R	0.633796	0.015077
Cerebellum 9 L	0.55506	0
Cerebellum 9 R	0.631055	0
Vermis 4/5	0.496548	0.043261
Vermis 6	0.683938	0.010133
Vermis 7	0.613539	0.020725
Vermis 8	0.478712	0
Vermis 9	0.507643	0

Table S17. Correlations and Dice's coefficients generated between ROIs of the OT and 1000FC datasets. ROIs = regions of interest. OT = orthostatic tremor. 1000FC = 1000 functional connectomes.

Table S18

Resting-State Functional Connectivity Results for Small Volume ROIs

					MNI Coordinates			
**Seed**	#	**k**	**T**	**v**	**x**	**y**	**z**	**Region(s)**
**PT**	1	163	17.692	+	7	-35	-33	Left cerebellar cortex
	2	21	6.553	-	11	-81	37	Right superior parietal lobule
**Right VIM**	1	314	34.842	+	11	-15	-1	Right thalamus proper
								Right ventral midbrain
	2	46	7.325	+	-9	-9	7	Left thalamus proper
	3	22	11.003	+	-7	-25	3	Left thalamus proper
**Left VIM**	1	270	44.32	+	-11	-13	-1	Left thalamus proper
								Left ventral midbrain
	2	56	7.91	+	33	23	3	Right insula
								Right pars triangularis
								Right pars opercularis
	3	47	6.43	-	-1	-85	-11	Bilateral lingual gyrus
								Left cerebellar cortex
	4	27	5.852	+	9	19	35	Right caudal anterior cingulate
	5	26	8.025	+	-65	-23	21	Left supramarginal gyrus
								Left postcentral gyrus
								Left superior temporal gyrus
	6	26	8.23	-	19	-47	29	No GM
	7	23	5.812	-	19	-93	11	Right lateral occipital gyrus
	8	21	5.916	-	5	-47	7	Right cingulate gyrus (isthmus)

	9	19	6.495	+	3	-9	1	Bilateral thalamus proper

Table S18. Clusters of significant RSFC for the PT, right VIM, and left VIM including coordinates and structures of the brain each cluster is present in. RSFC = resting state functional connectivity. k = cluster size in voxels. T = peak T statistic in the cluster. V = positive or negative nature of cluster to seed. PT = pontine tegmentum. VIM = centralis intermedius of the thalamus.

Table S19

Resting-State Functional Connectivity Results for PCC

				MNI Coordinates			
**#**	**k**	**T**	**v**	**x**	**y**	**z**	**Region(s)**
**1**	5172	28.266	+	-1	-59	23	Bilateral precuneus
							Bilateral posterior cingulate
							Bilateral cingulate gyrus (isthmus)
							Left thalamus proper
							Right cuneus
							Left hippocampus
							Left paracentral lobule
**2**	1686	14.571	-	11	3	49	Bilateral superior frontal gyrus
							Bilateral caudal anterior cingulate
							Bilateral posterior cingulate
							Right precentral gyrus
							Bilateral paracentral lobule
**3**	1519	18.59	-	57	11	-3	Right insula
							Right precentral gyrus
							Right pars opercularis
							Right pars triangularis
							Right superior temporal gyrus
							Right putamen
							Right middle frontal gyrus (rostral)
							Right lateral orbitofrontal cortex

							Right postcentral gyrus
**4**	1501	13.169	+	-45	-67	35	Left inferior parietal lobule
							Left supramarginal gyrus
**5**	923	11.021	-	59	-21	15	Right supramarginal gyrus
							Right superior temporal gyrus
							Right postcentral gyrus
**6**	861	11.9	+	43	-67	41	Right inferior parietal lobule
**7**	830	12.082	+	-23	31	39	Left middle frontal gyrus (caudal)
							Left middle frontal gyrus (rostral)
							Left superior frontal gyrus
**8**	649	14.497	-	-55	7	1	Left precentral gyrus
							Left insula
							Left pars opercularis
							Left superior temporal gyrus
							Left postcentral gyrus
**9**	438	11.726	-	-63	-25	19	Left supramarginal gyrus
							Left superior temporal gyrus
							Left postcentral gyrus
**10**	418	14.49	+	27	31	53	Right superior frontal gyrus
							Right middle frontal gyrus (caudal)
**11**	365	9.404	+	-61	-7	-15	Left middle temporal gyrus
							Left superior temporal gyrus
**12**	234	9.051	+	7	57	-9	Bilateral medial orbitofrontal cortex
							Bilateral superior frontal gyrus

							Left frontal pole
**13**	223	12.571	-	-39	51	23	Left middle frontal gyrus (rostral)
**14**	208	9.397	-	41	41	33	Right middle frontal gyrus (rostral)
**15**	167	8.528	+	63	-17	-13	Right middle temporal gyrus
**16**	141	7.638	+	-9	55	7	Bilateral superior frontal gyrus
**17**	135	10.492	-	-25	-67	-21	Left cerebellar cortex
							Left lingual gyrus
							Left fusiform gyrus
**18**	76	7.475	+	-11	55	39	Left superior frontal gyrus
							Left middle frontal gyrus (rostral)
**19**	72	8.025	+	-31	-79	-31	Left cerebellar cortex
**20**	71	7.192	-	53	-49	-3	No GM
**21**	69	9.342	-	-41	-7	-11	Left insula
							Left superior temporal gyrus
**22**	66	6.237	+	39	-75	-37	Right cerebellar cortex
**23**	61	6.722	-	29	-65	-19	Right cerebellar cortex
							Right fusiform gyrus
**24**	60	8.072	+	-29	-19	-17	Left hippocampus
**25**	58	6.299	+	-3	-59	-47	Bilateral cerebellar cortex
**26**	37	7.794	+	-25	-31	-15	Left hippocampus
							Left parahippocampal gyrus
**27**	34	6.721	-	-31	-85	17	Left inferior parietal lobule
**28**	33	5.624	-	51	3	47	Right precentral gyrus
							Right postcentral gyrus
**29**	32	7.101	+	49	-57	-39	Right cerebellar cortex
**30**	28	6.5	-	-31	31	9	No GM

**31**	27	7.633	-	-35	-51	-29	Left cerebellar cortex
**32**	20	6.304	-	41	-43	55	Right superior parietal lobule
**33**	19	6.117	+	5	-49	-51	Left middle temporal gyrus
							Left superior temporal sulcus (bank)
**34**	19	7.048	-	-63	-53	7	Right cerebellar cortex
**35**	18	7.571	-	-45	-77	-11	Left precentral gyrus
**36**	18	5.84	-	-51	-5	51	Left lateral occipital gyrus

Table S19. Clusters of significant RSFC for the PCC including coordinates and structures of the brain each cluster is present in. PCC = posterior cingulate cortex. RSFC = resting state functional connectivity. k = cluster size in voxels. T = peak T statistic in the cluster. V = positive or negative nature of cluster to seed.
